# Short Vacation Improves Stress-Level and Well-Being in German-Speaking Middle-Managers—A Randomized Controlled Trial

**DOI:** 10.3390/ijerph15010130

**Published:** 2018-01-13

**Authors:** Cornelia Blank, Katharina Gatterer, Veronika Leichtfried, Doris Pollhammer, Maria Mair-Raggautz, Stefan Duschek, Egon Humpeler, Wolfgang Schobersberger

**Affiliations:** 1 Department of Psychology and Medical Sciences, Institute of Sports Medicine, Alpine Medicine & Health Tourism, UMIT, 6060 Hall in Tirol, Austria; katharina.gatterer@umit.at (K.G.); info@alpinespiritcenter.at (V.L.); doris1711@hotmail.com (D.P.); info@loesungsschmiede.at (M.M.-R.); wolfgang.schobersberger@tirol-kliniken.at (W.S.); 2 Department of Psychology and Medical Sciences, Institute of Psychology, UMIT, 6060 Hall in Tirol, Austria; stefan.duschek@umit.at; 3 IHS Forschungsinstitut für Urlaubs- und Freizeitmedizin Sowie Gesundheitstourimsus, 6900 Bregenz, Austria; egon.humpeler@welltain.at; 4 Institute of Sports Medicine, Alpine Medicine & Health Tourism, Tirol Kliniken GmbH, 6020 Innsbruck, Austria

**Keywords:** Health Tourism, well-being, recovery, middle management

## Abstract

Stress in the work place has a detrimental effect on people’s health. Sufficient recovery is necessary to counteract severe chronic negative load reactions. Previous research has shown that vacationing for at least seven consecutive days provided an efficient recovery strategy. Yet, thus far, the effects of short vacations and the mode of vacation (whether at home or in a new environment) have rarely been studied. We investigated the immediate and long-term effects of a short vacation (four nights) on well-being and perceived stress and whether the mode of vacation impacted on these results. Data was obtained from 40 middle managers (67.5% men and 32.5% women). The intervention group (*n* = 20) spent a short vacation in a hotel outside their usual environment. The control group (*n* = 20) spent their vacation at home. Results indicated that one single short-term vacation, independent of the mode, has large, positive and immediate effects on perceived stress, recovery, strain, and well-being. Strain levels decreased to a greater extent in the intervention group compared to the control group. The effects can still be detected at 30 days (recovery) and 45 days (well-being and strain) post-vacation. Encouraging middle management employees to take short vacations seems to be an efficient health promotion strategy; environmental effects seem to play a minor role.

## 1. Introduction

Stress, a potential danger to a person’s health and well-being, is a “product of the transaction between an individual and its environment” [1]. It is commonly experienced negatively if perceived strain outweighs available coping resources [2]. The work place is considered as a source of strain that causes stress leading to detrimental health effects [3,4,5]. Evidence shows that workers and executives in middle management positions are more susceptible to increased stress levels compared to, for example, top managers [6,7,8,9,10]. Based on the effort–recovery model (ERM) [11], the workplace is associated with effort expenditures that can develop into severe and chronic load reactions if recovery is insufficient or incomplete. Thus, recovery as a process of “psychophysiological unwinding” [12] appears to play a major role in stress reduction and can act as an intervention in the development of long-term negative health reactions to normal stressors at work [12].

Leisure and free time, a special form of recovery, has the potential to distract people from stress [3,4,5] and enhance their positive mood [13]. Examples of leisure time activities that exert positive effects on stress include socialization, fun and laughter, and travel [14] as well as physical activities and high quality sleep [15]. In view of the need for sufficient recovery to counter the long-term effects of stress, travel and vacation as recovery strategies have received much attention; positive effects on the well-being of different target groups were found [16,17,18,19]. In subjects with high job-related stress levels, positive health effects from a one-week active vacation were reported [20]. However, the positive effects ascribed to vacations wane after a period of a couple of days to three or four weeks after resuming work [16,17,18,20,21].

Published research has mostly investigated the effects of a long-term vacation over a period of at least seven consecutive days. Yet—based on the limits of employees’ time resources and a higher workload—a new direction in tourism diverges from the traditional long-term vacation towards a shorter, more intensive period of experience and recovery [22]. Statistics on the average lengths of stay of all tourists in Austria indicate a decrease from 6.2 days in 1980 to 3.6 days in 2012 [23]. We are aware of only one study up until now that examined the immediate and long-term effects of a short vacation on workers’ health and well-being [24]. Although their results suggested that even short vacations lasting less than five days improve employees’ health and well-being, research on this topic is still limited and several questions remain unanswered. It is still unclear which mechanisms lead to the improvements (specific activities during vacation), how sustainable the positive effects of short vacations are, and whether these findings can be transferred to target groups at risk other than workers. Additionally, various authors also discussed whether the mode of vacation might affect the size of the positive health effects of a vacation. It is argued that “simply being away” and changing the “normal environment” eventually facilitates the recovery process [25]. This assumes that traveling outside one’s usual environment during vacation time to escape daily routines is perceived as effective stress relief [26,27]. However, to the best of our knowledge, no research has addressed this question experimentally so far.

The aim of this study is therefore to address the following research questions: Does one short vacation have positive effects on the well-being and stress of middle managers? How sustainable are its potential effects? Does the mode of vacation impact the effects on well-being and stress?

## 2. Materials and Methods

### 2.1. Design and Procedure

**Figure 1:**
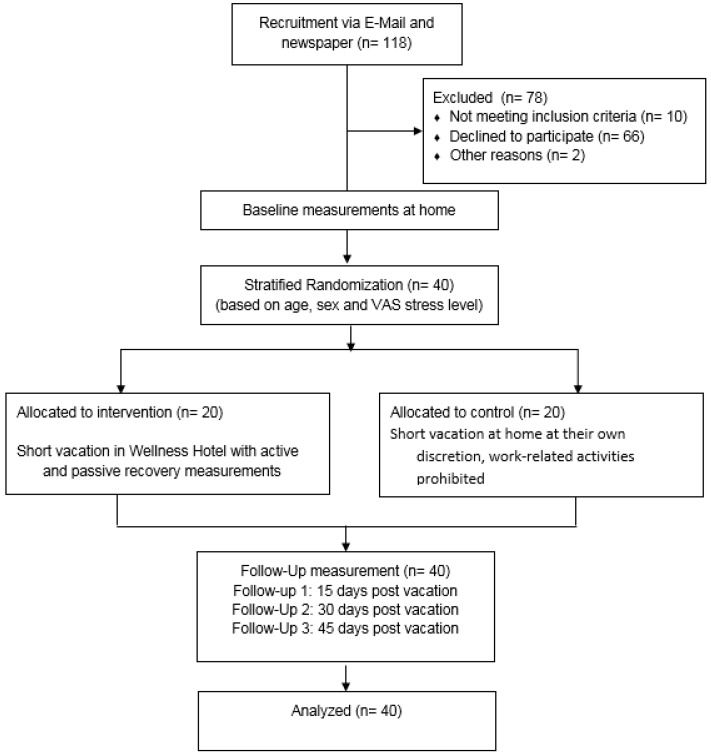
Flow diagram of data collection and participant flow.

Participants in this randomized, controlled, two-armed intervention study were matched (stratified randomization) in either the experimental (short vacation, four nights) or control (four nights free time at home away from work) group based on age, gender and stress level based on a Visual Analogue Scale (VAS). To minimize a possible bias affecting subjective well-being from the joyful anticipation of going on vacation, participants were matched after they filled in the baseline questionnaires (“Questionnaire for Recuperation and Strain” [Erholungs-Belastungs-Fragebogen] (EBF), “Questionnaire for general well-being” [Fragebogen zum Allgemeinen Wohlbefinden] (FAHW), “Perceived Stress Questionnaire” (PSQ)). The baseline measurement (BEX) was dependent on the point in time at which the participant agreed to take part in the study but was at least four weeks prior to the vacation (time off) for all participants. Both groups followed the same study protocol in terms of questionnaire measurements. Due to its high sensitivity, participants filled in the EBF and FAHW questionnaires on the first (T1) and last day (T2) of their time off work. Time points of follow-up measures were determined based on previous research with a focus on the sustainable effects of long-term vacation [18]; questionnaires were filled in 15 days (follow-up (FU) 1), 30 days (FU 2), and 45 days (FU 3) after returning to work. The less sensitive PSQ was filled in three times in total, firstly at BEX, at the end of the vacation (T2), and again at FU 1. The detailed study procedure is outlined in Figure 1. In addition, the CONSORT 2010 checklist is available as supplementary material Table S1.

This study has received ethical approval from the ethics committee of the Medical University of Innsbruck (UN2013-0029). Participation was voluntary, and all participants provided written informed consent prior to the study.

### 2.2. Participants

Middle managers with an elevated stress-level from different companies included in this study were recruited through invitations to large institutions. Middle managers were defined as having managerial tasks including personnel responsibilities but not being the chief executive officer and/or head of a company. Interested participants were screened according to the following inclusion and exclusion criteria based on a completed socio-demographic information sheet prior to inclusion in the study. Inclusion criteria consisted of a subjective stress level between 5 and 9 (based on a VAS ranging from 1–10), an age of between 25 and 64 years, employment as an executive in middle management, and availability for an initial examination and a four-day vacation. Exclusion criteria included having a private vacation planned between the confirmation of participation and the intervention (as well as in the 45-day period following the intervention), the presence of severe neurological and psychiatric diseases, and all acute diseases shortly before or during the intervention. Finally, a travel time of more than three hours to the hotel accommodation led to exclusion as the literature states that long-distance travel can have negative effects on an individual’s health [28].

### 2.3. Experimental Conditions

As proposed elsewhere [24], the short vacation was operationalized as a long weekend (four full days, Thursday to Sunday with the arrival on Wednesday evening) in a Tyrolean Wellness Hotel. Participants paid 150 €/stay, the rest of the costs were covered by the study budget. Family members were not allowed to travel with the participants. Based on findings around the positive effects of physical activity and recovery [15], participants had to partake in one session of moderate physical activity (Nordic walking, swimming) and one session of active recovery (Yoga or Qui-Gong). Activities were led by a certified coach from the hotel and exclusively offered to the participants. The remaining time at the hotel could be used at the participants’ own discretion.

The control group spent the same amount of time at home and was instructed to partake in activities they would normally do in their free time but were not allowed to undertake work-related activities.

### 2.4. Measurements

#### 2.4.1. Well-Being

To assess well-being, the German FAHW [29] was used. It subsumes three facets of well-being: psychological (emotions and autonomy), physiological (vitality and concentration), and social (social contacts) well-being under one score, calculated by the mean value of 42 items answered on a 5-point Likert scale (1 = *definitely not* to 5 = *definitely so*). Cronbach’s alpha was acceptable at all times of measurement with *α*: T1 = 0.82, T2 = 0.84, FU1 = 0.83, FU2 = 0.84, FU3 = 0.86.

#### 2.4.2. Recovery and Strain

As slow unwinding and low sleep quality are examples of symptoms of insufficient recovery, subjective recovery complaints and subjective strain were assessed by means of the German version of the “Questionnaire for Recuperation and Strain”—the EBF-B [30]. It covers seven different subtests of strain (general-, emotional-, social- and somatic strain, conflict, fatigue, and lack of energy) as well as five subtests of recovery (success, sleep, social-, somatic- and general recovery). The questionnaire consists of 24 items answered on a 7-point Likert scale (0 = *never* to 6 = *always*). The test–retest reliability after 24 h was reportedly between 0.79 and 0.91 [30]. Cronbach’s alpha for strain was acceptable with *α_strain_:* T1 = 0.83, T2 = 0.81, FU1 = 0.89, FU2 = 0.83, FU3 = 0.88 and *α_recovery_:* T1 = 0.78, T2 = 0.71, FU1 = 0.86, FU2 = 0.77, FU3 = 0.83.

#### 2.4.3. Perceived Stress

The short German version [31] of the PSQ [32] was used to assess perceived stress. It consists of 20 items answered on a 4-point Likert scale (1 = *almost never* to 4 = *often*). The final scale ranges between 0 and 100, based on a linear transformation. No test–retest reliability is available for this test; however, the split-half reliability was reportedly between >0.70 and >0.80 [31]. Cronbach’s alpha was acceptable with *α:* BEX = 0.70, T2 = 0.73, FU1 = 0.70.

### 2.5. Statistical Analyses

To analyze the immediate effects of the short vacation, two-factor-analyses of variance (ANOVA) with repeated measurements on two occasions (the beginning of the vacation and the end of the vacation) were performed including the dependent variables of well-being, perceived stress, strain, and recovery. Gender was included as an additional factor to analyze perceived stress, as the literature indicates a possible gender-effect. Main time effects and interaction effects were analyzed to identify differences between the intervention and control groups. Where appropriate, data was subsequently analyzed separately for the control and intervention groups using a one-factor-ANOVA. To assess the sustainability of possible effects, two-factor-analyses of variance (ANOVA) with repeated measurements on four, respectively three (PSQ) occasions (beginning of the vacation, 15, 30, and 45 days post vacation) were performed including the dependent variables well-being, perceived stress, strain, and recovery. Gender was included as an additional factor to analyze perceived stress, as the literature indicates a possible gender-effect. Main time effects were assessed using Bonferroni-corrected post-hoc tests. If appropriate, data from the two groups was subsequently analyzed separately using a one-factor-ANOVA. The significance level was set at *p* < 0.05.

## 3. Results

Data was obtained from a sample of 40 executives in middle management positions. Socio-demographic characteristics and subjective stress levels of the study population, comprising 27 men (67.5%) and 13 women (32.5%), are presented in Table 1. In total, 20% of the participants reported that they regularly took medications for hypertension, high blood lipids, or thyroid dysfunction. A small proportion of participants (2.5%; *n* = 1) suffered from chronic headache, 10.0% (*n* = 4) from sleep disturbances, and 5.0% (*n* = 2) had hypertension.

A successful randomization was confirmed by analyses of the baseline values of the matching criteria (age: *p* = 0.28; gender: *p* = 0.74; stress level: *p* = 0.42). In addition, all four outcome variables (well-being, recovery, strain, and perceived stress) were comparable between the groups (0.92 > *p* > 0.44). Perceived stress (PS) showed significantly different values between men and women (females: 53.7 ± 13.0 vs. males: 39.14 ± 13.3; *p* = 0.002) but no association with age (*r* = −0.07; *p* = 0.69). Therefore, we added gender as an additional factor to the ANOVA analyzing perceived stress.

### 3.1. Immediate Effects of a Short-Term Vacation on Well-Being, Recovery, Strain, and Perceived Stress-Level

Well-being and recovery significantly increased; strain and perceived stress significantly decreased over time in both groups. No significant interaction effects were found for well-being, recovery, and perceived stress-level; interaction effects were found for strain. A post-hoc independent *t*-test of delta strain indicated that strain decreased more in the intervention group than in the control group (−1.3 ± 0.4 vs. −0.9 ± 0.6, *p* = 0.02). Detailed results are presented in Table 2.

Concerning the perceived stress level, results additionally indicated a between-subject gender effect (*p* = 0.001) with females having a significantly higher stress level, but there was no gender-specific interaction effect (*p* = 0.65).

### 3.2. Sustainability of the Effects of a Short-Term Vacation on Well-Being, Recovery, Strain, and Perceived Stress-Level

Well-being and recovery significantly increased; strain and perceived stress significantly decreased over time in both groups. Post-hoc tests showed that recovery values were still at an improved level 30 days post-vacation. Well-being and strain values remained at an improved level until 45 days post-vacation. No interaction effects were found for any of the variables.

**Figure 2:**
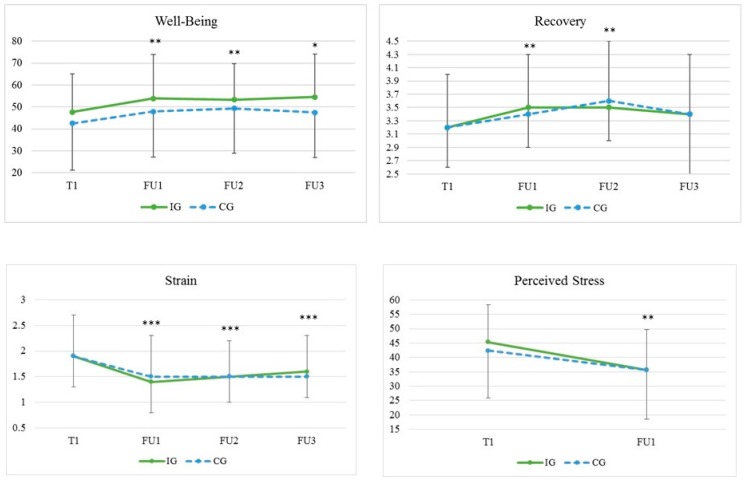
Sustainability of the effects of a short-term vacation on well-being, recovery, strain, and perceived stress. T1: Beginning of vacation, FU1: 15 days post-vacation, FU2: 30 days post-vacation, FU3: 45 days post-vacation. Control: Four nights at home; Intervention: Four nights in a 4* hotel. *** *p* < 0.001, Bonferroni-corrected, compared to T1. ** *p* < 0.01, Bonferroni-corrected, compared to T1.

Concerning the perceived stress level, a significant between-subject gender effect (*p* = 0.002) was found, but no gender-specific interaction effect was observed (*p* = 0.63). Detailed results are presented in Figure 2.

## 4. Discussion

The present study investigated the immediate effects and their sustainability of one short vacation of four consecutive days on aspects of perceived stress, strain, recovery, and well-being, including the impact of the mode of vacation. Data suggests that a short-term vacation, independent of the mode of vacation, might have positive immediate effects on perceived stress, recovery, strain, and well-being. However, there was a more marked decrease in the strain values of the hotel group compared to those of the control group. Improvements in perceived stress were maintained until day 15, as well as recovery until day 30 post-vacation. Well-being and strain levels remained significantly improved for 45 days post-vacation with no difference between the groups. Nonetheless, results should be generalized with caution due to (a) a missing control group from the work environment and (b) a very selective vacation program.

### 4.1. Immediate Effects of One Short-Term Vacation

Decreasing the level of strain and increasing recovery capacity are both important factors in coping with everyday stressors [29]. Results of our study suggest that even a short-term vacation might have beneficial effects on one’s well-being, strain level, and stress. The effects’ sizes suggest that people benefited most with regards to their strain level, which is important for protecting oneself against the long-term negative effects of stress. According to Lazarus and Folkman [2], negative stress experience is a result of lacking coping resources while facing daily strain. Reducing strain is therefore one possibility for reducing negative stress experiences, along with increasing coping resources.

Female participants in this study displayed a significantly higher perceived stress level than male participants. This is in line with previous research in that women tend to report a higher stress level [33] and show different stress coping strategies [14]. Even though males are reported to be generally more positive about leisure travel as a stress-coping strategy [14], we did not find any differences between genders in the beneficial effects of a short vacation.

### 4.2. Sustainability of Effects of One Short-Term Vacation

The immediate effects of vacations, no matter if short- or long-term, were generally positive [16,18,24], but faded out very quickly [18,20,21]. The most interesting finding of this study was the sustainability of positive effects, which were still present after 15 and 30 days for perceived stress and recovery and even 45 days post-vacation for well-being and strain. It seems that for employees in management positions, a short-term vacation can already sustainably benefit their perceived well-being, strain, and recovery level. Explanations for these findings might be two-fold: first, Fritz and Sonnentag [21] explain that the quick fade out after a long vacation may be due to returning to a massive pile of work. Thus, a shorter vacation might prolong the long-term effectiveness by not having to return to a comparably high work-load but still being away long enough to fully recover from daily work-related stressors. A second explanation might be the activities included in the vacation. Strauss-Blasche and Ekmekcioglu [34] and Strauss-Blasche and Reithofer [19] suggested that physical activity during vacations might have the potential to prolong positive vacation effects. In sum, it seems that short vacations of four nights are long enough to fully recover and short enough to limit the massive pile of work after return, which possibly causes a quick fade-out. However, it must also be considered that even though we could show positive effects 45 days after the vacation, this timescale is still short in comparison to the long timescale of the working life. Therefore, short vacations might be a reasonable choice to buffer daily stressors at work, although the more lasting preventive measure would probably still be the reduction of stress at the source.

### 4.3. Mode of Vacation

Regarding the effect on recovery based on the mode of vacation, we could barely find evidence for the proposed assumptions of de Bloom and Geurts [25] and Davidson and Eden [35] that escaping daily routines by travelling outside the usual environment has an additional impact on vacation effects. Strain was the only variable that differed in its development between the groups and only with regards to the immediate effects. A short-term vacation away from home can therefore be recommended to reduce perceived strain, especially if there are insufficient coping resources. The reduced strain level might also explain why escaping daily routines is perceived as stress relief [26].

Evidence suggested that positive vacation effects were prolonged by including physical activity [19,34,36]. Even though only the Intervention Group (IG) had to follow a specific activity program during their vacation, non-findings of interaction effects in most of the variables regarding immediate and long-term effects of vacation might be explained with the fact that the control group might have taken the opportunity to be physically active and relax in their free time as well. The control group was only instructed not to undertake any work-related activity. Another explanation might be that a short vacation of four nights is too short to show the effects of a specific vacation program. Thirdly, the effects of almost all variables still show significant improvement after 45 days, which is longer than in most other research [18]. Therefore, it is also plausible that we have a ceiling effect and that an interaction effect would not be apparent in the data until after the 45-day period. Future research might want to include post-hoc measures after this point. Finally, escaping the usual environment might not have an effect over and above the vacation experience, at least not regarding well-being and recovery.

## 5. Conclusions

One single short vacation of four nights has positive effects on the well-being, recovery, strain, and perceived stress of middle managers, independent of the mode of vacation. These effects are measurable even 30 days (recovery) and 45 days (well-being and strain) after the end of the short vacation. Given the statistics on the decreased average length of stay of all tourists in Austria [23], a short vacation might be a worthwhile alternative for protecting the health of middle managers, even though reducing stress at the source should not be ignored. We could provide some evidence that this vacation length appears to be sufficient to recover from stress, a finding that might be even more relevant in countries where the yearly vacation time is lower than in European or Scandinavian countries (the United States with an average of 3.9 weeks of paid vacation including public holidays [37]). Yet, it should be kept in mind that we introduced a very selective vacation program which did not allow any family members to join and included physical activity as well as active recovery. Even though it seems somewhat unusual at this point, short vacations of this type could possibly be integrated into an occupational health prevention catalogue, and as such become a usual procedure. Nevertheless, until confirmed in further studies, results should be generalized with caution.

Overall, the expected additional positive effects of escaping daily routines and the usual environment do not seem to be crucial for recovery. Nevertheless, encouraging middle management employees to take short vacations seems to be an efficient workplace health promotion strategy to decrease stress and strain and increase their well-being and recovery—factors that have been shown to protect against long-term negative health effects.

Some limitations of this study need to be acknowledged. The activities of the control group during time off were not recorded, and could have provided relevant information towards explaining the missing interaction effects between the two groups. The control group was under a control condition based on the WTO tourism definition of being away from one’s usual environment. No control group in working conditions was included, thus alternative explorations cannot be completely ruled out. Additionally, improvements in the outcome variables could also have been due to limitations in the repeated measurements and scales chosen. We tried to minimize this bias by selecting well-validated and commonly used scales to assess the outcome variables. Reported test–retest reliabilities were satisfactory. Moreover, based on previous research, one can assume that working in a middle management position might be a rather onerous condition. Feelings of group membership within the intervention group who spent four nights together in one hotel might have biased the results of the questionnaires in either direction. Finally, even though they were instructed not to, we cannot be certain that participants did not have another vacation during the time of follow-up appointments nor did we record the exact activities of the participants during the 45 days post-vacation.

## Figures and Tables

**Table 1:** Socio-demographic characteristics of study sample groups.

	Control Group (*n* = 20)	Intervention Group (*n* = 20)	*p*
	M ± SD	M ± SD	
Age	46.80 ± 8.11	44.00 ± 8.05	0.28
Height (cm)	174.95 ± 8.43	176.00 ± 7.59	0.68
Weight (kg)	76.80 ± 12.53	75.95 ± 14.70	0.85
BMI	25.01 ± 3.32	24.39 ± 3.77	0.59
PSL ^1^	7.31 ± 1.07	7.55 ± 0.76	0.42

^1^ PSL: perceived stress level based on the Visual Analogue Scale (VAS).

**Table 2:** Immediate effects of vacation on well-being, recovery, strain, and perceived stress.

	IG			CG			All			Time Effect (η ^2^)	Interaction Effect (η ^2^)
	*n*	T1 ^1^	T2 ^2^	*n*	T1	T2	*n*	T1	T2		
Well-being	19	47.3 ± 16.9	59.1 ± 21.3	19	42.8 ± 21.3	49.9 ± 18.2	38	45.1 ± 19.1	54.8 ± 19.9	<0.001 (0.43)	ns ^4^ (0.04)
Recovery	20	3.2 ± 0.6	3.5 ± 0.6	20	3.2 ± 0.8	3.7 ± 0.9	40	3.2 ± 0.7	3.6 ± 0.7	<0.001 (0.41)	ns (0.01)
Strain	20	1.9 ± 0.5	0.7 ± 0.3	20	1.9 ± 0.8	1.0 ± 0.7	40	1.9 ± 0.7	0.9 ± 0.5	<0.001 (0.83)	<0.01 (0.13)
Perceived Stress ^3^	20	45.3 ± 13.2	36.2 ± 17.4	20	42.4 ± 16.4	35.5 ± 16.1	40	43.9 ± 14.7	35.8 ± 16.6	<0.01 (0.25)	Ns (0.005)

^1^ T1: Start of vacation, ^2^ T2: End of vacation, ^3^: T1 = Baseline value; ^4^ ns: not significant. IG: Intervention Group; CG: Control Group.

## Supplementary Materials

The following is available online at www.mdpi.com/1660-4601/15/1/130/s1, Table S1: CONSORT 2010 checklist of information to include when reporting a randomised trial.
